# Evolutionary origin and function of NOX4-art, an arthropod specific NADPH oxidase

**DOI:** 10.1186/s12862-017-0940-0

**Published:** 2017-03-29

**Authors:** Ana Caroline Paiva Gandara, André Torres, Ana Cristina Bahia, Pedro L. Oliveira, Renata Schama

**Affiliations:** 1 Instituto de Bioquímica Médica Leopoldo de Meis, Universidade Federal do Rio de Janeiro, Rio de Janeiro, Brazil; 2 Laboratório de Biologia Computacional e Sistemas, Instituto Oswaldo Cruz, Fiocruz, Rio de Janeiro, Brazil; 3 Instituto de Biofísica, Universidade Federal do Rio de Janeiro, Rio de Janeiro, Brazil; 4 Instituto Nacional de Ciência e Tecnologia em Entomologia Molecular – INCT-EM, Rio de Janeiro, Brazil

**Keywords:** NADPH oxidase, Gene loss, Gene family, Arthropods, ROS, Reactive oxygen species

## Abstract

**Background:**

NADPH oxidases (NOX) are ROS producing enzymes that perform essential roles in cell physiology, including cell signaling and antimicrobial defense. This gene family is present in most eukaryotes, suggesting a common ancestor. To date, only a limited number of phylogenetic studies of metazoan NOXes have been performed, with few arthropod genes. In arthropods, only NOX5 and DUOX genes have been found and a gene called NOXm was found in mosquitoes but its origin and function has not been examined. In this study, we analyzed the evolution of this gene family in arthropods. A thorough search of genomes and transcriptomes was performed enabling us to browse most branches of arthropod phylogeny.

**Results:**

We have found that the subfamilies NOX5 and DUOX are present in all arthropod groups. We also show that a NOX gene, closely related to NOX4 and previously found only in mosquitoes (NOXm), can also be found in other taxonomic groups, leading us to rename it as NOX4-art. Although the accessory protein p22-*phox,* essential for NOX1-4 activation, was not found in any of the arthropods studied, NOX4-art of *Aedes aegypti* encodes an active protein that produces H_2_O_2_. Although NOX4-art has been lost in a number of arthropod lineages, it has all the domains and many signature residues and motifs necessary for ROS production and, when silenced, H_2_O_2_ production is considerably diminished in *A. aegypti* cells.

**Conclusions:**

Combining all bioinformatic analyses and laboratory work we have reached interesting conclusions regarding arthropod NOX gene family evolution. NOX5 and DUOX are present in all arthropod lineages but it seems that a NOX2-like gene was lost in the ancestral lineage leading to Ecdysozoa. The NOX4-art gene originated from a NOX4-like ancestor and is functional. Although no p22-*phox* was observed in arthropods, there was no evidence of neo-functionalization and this gene probably produces H_2_O_2_ as in other metazoan NOX4 genes. Although functional and present in the genomes of many species, NOX4-art was lost in a number of arthropod lineages.

**Electronic supplementary material:**

The online version of this article (doi:10.1186/s12862-017-0940-0) contains supplementary material, which is available to authorized users.

## Background

Reactive oxygen species (ROS) are generated by the partial reduction of oxygen, producing a number of short-lived and highly electrophilic molecules (eg. superoxide anion (^**.**^O_2_^−^), hydrogen peroxide (H_2_O_2_), among others). These molecules have originally been seen as deleterious but in the last decade a wide array of diverse biochemical functions were assigned to them in several organisms. ROS are implicated in important basic biological processes that include cell differentiation, development and motility, cytoskeletal reorganization, cell survival and apoptosis, stress response, gut homeostasis and defense, cell signaling and transcriptional regulation [1–3]. In *Drosophila melanogaster*, for example, the ROS producing dual oxidase gene (DUOX) seems to play a critical role in gut innate immunity [4]. ROS can be produced in different ways: (i) as a side reaction of common enzymatic activity, (ii) exogenous compound induction but also (iii) as a physiological response produced by specialized enzymes. The gene family known as NADPH oxidase (NOX) is responsible for the physiological production of ROS [2, 5]. This NOX-dependent generation of ROS is highly conserved across virtually all multicellular forms of life.

Metazoan NOXes are divided in three subfamilies (NOX1-4, NOX5 and DUOXes). All proteins have two canonical domains that are also shared with ferric reductase enzymes: a heme-containing transmembrane domain and a C-terminal cytoplasmic dehydrogenase (DH) domain, which contains FAD and NADPH-binding sites [6, 7]. The ferric reductase domain is characterized by 6 transmembrane α-helices (TM1-6) where four conserved histidine residues (two on helix 3 and two on helix 5) bind two heme molecules. The electrons are transferred from NADPH to FAD, then to the heme molecules and, finally, to molecular oxygen (O_2_) which becomes superoxide by partial reduction. Besides the canonical domains, members of the DUOX subfamily also have at least two EF-hand calcium-binding domains and one N-terminal peroxidase-like domain [8, 9] while NOX5 enzymes have four N-terminal EF-hand calcium binding domains [8, 10–13].

During the course of evolution, individual NOXes have acquired different regulatory systems [6, 14, 15] as separate cytosolic and membrane bound subunits [7]. The activation of NOX2 has been extensively studied since point mutations in this gene cause the X-linked chronic granulomatous disease (CGD) [16]. This was the first NOX gene described and, in mammals, it is expressed in phagocytic cells, producing superoxide [2, 17, 18]. However, ROS are also produced in a variety of other cell types and tissues [1, 19]. In the NOX1-4 subfamily, all proteins need to be associated with a non-glycosylated integral protein called p22-*phox* to be active [6, 12, 15]. This protein has a cytoplasmic proline-rich region (PRR) that helps stabilize the NOX enzymes at the membrane [20, 21]. NOX1-3 need other cytosolic proteins and NOX2 and NOX3 also need the small GTPase Rac to function [22–25]. NOX4 enzymes seem to require only p22-*phox* for basal ROS production [5, 14, 21, 26]. NOX4 is constitutively active in the presence of p22-*phox* [26] simply because the conformation of its DH domain seems to allow the transfer of electrons from NADPH to FAD [27]. In the other two subfamilies, NOX5 and DUOX1-2, apart from other proteins, calcium molecules are important for ROS production since they are needed for enzyme activation [10, 15, 28–31].

In mammals, the most widely studied group so far, there are seven genes (NOX1-4, NOX5 and DUOX1-2) that belong to the different subfamilies of NOXes. The presence of NOX genes in most eukaryotic groups suggests a common ancestor early in evolution with patterns of expansion and gene loss [6, 7]. In metazoa, NOX1-4 seem to have emerged from ancestral EF-hand containing subfamilies (NOX5 and DUOX) [7]. The relationship among the genes within the NOX1-4 subfamily is somewhat unclear. NOX2 is present in most groups and was suggested as the ancestral NOX1-4 in animals [32], however, it has been lost in Ecdysozoa (phyla Nematoda and Arthropoda). Previous studies suggested that NOX4 appeared in the deuterostomes (although not in Echinodermata) but, recently, this gene was found in the genome of the sea anemone *Nematostella vectensis* which indicates an earlier divergence than previously thought [7]. NOX1 seems to be present only in vertebrates and NOX3 only in mammals and reptiles/birds [32]. In arthropods, only NOX5 and DUOX genes have been found [32]. Interestingly, a new gene called NOXm was found only in mosquitoes but its origin has not been carefully examined [7, 32].

To date, only a limited number of phylogenetic studies of metazoan NOXes have been performed where just a few available arthropod genes (from the species *Drosophila melanogaster*, *Apis mellifera* and *Anopheles gambiae*) were utilized [6, 7, 32]. The most recent study used a whole superfamily approach with deep branching nodes but again, only a few metazoan species were analyzed [7]. The NOXm gene described for mosquitoes, for example, has been linked to their hematophagous habit [32] and warrants attention. Despite its ecological importance and relevance to vector biology, its evolutionary history and functionality have never been looked at. We only know that *Wolbachia* infection in *Aedes aegypti* leads to increased NOXm and DUOX transcript levels and their silencing suppresses the expression of some antimicrobial peptides [33]. More recently, Park et al. 2015 also found that eicosanoids seem to mediate ROS production by a NOX4-like gene in the moth *Spodoptera exigua* [34].

Given the role of NOXes in insects and how little is known about their evolution in this wide and diverse group of animals, a deeper understanding of their phylogenetic relationship is needed. Furthermore, the discovery of the NOXm gene raises intriguing questions about its function and evolution in arthropods [32]. Here, we performed a more extensive analysis that profited by the availability of several recently sequenced arthropod genomes to characterize the evolutionary history of the NOX family in arthropods and the structural and functional features of NOXm. We performed a thorough search of genomes and transcriptomes, when available, and were able to browse most branches of arthropod phylogeny. Furthermore, through bioinformatics and, to some extent, experimental work we show that the functionality features necessary for ROS production are present in NOXm, a gene closely related to vertebrate NOX4. Finally, we determined that this gene is not restricted to mosquitoes and is present in a number of arthropod lineages although it has also been lost in many. Since NOXm is not limited to Culicidae (mosquitoes) but present in a number of arthropod lineages, we suggest renaming it to NOX4-art.

## Results

### Gene searches

Sequences encoding putative NOX genes were identified from the predicted protein set of 70 arthropods, one choanoflagellate, two cnidarians, one sponge, four mollusks, two annelids, six nematodes, one echinoderm, one cephalochordate, one urochordate, and eight vertebrate genomes [see Additional file 1]. This search greatly improves on previous searches of NOX genes in invertebrate genomes, especially nematodes, mollusks, annelids and arthropods; the latter being the main focus of this work. The searches and posterior gene structure analysis of all genes in all genomes (95 in total) revealed a total of 113 DUOX, 94 NOX5, 34 NOX4/NOX4-art, 6 NOX1, 3 NOX3 and 21 NOX2 genes. Additional file 1 summarizes the organisms analyzed and number of copies of NOX genes found in each genome and their source [see Additional file 1]. We found that in only four cases the automated genome predictions did not recover one or more of the NOX genes expected for the species and these were found with tBLASTn and Exonerate searches of the scaffolds. Four new genes were predicted using GeneWise: DUOX genes from the species *Eurytemora affinis* (Scaffold63, region 576882-539914) and *Blatella germanica* (Scaffold551, region 278933-220027) and NOX5 genes from the species *Phlebotomus papatasi* (scaffold:PpapN1:Scaffold23814, region 33-1651) and *Heliconius melpomene* (HE671948.1, region 63215-107001).

In public domain databases, arthropod genes that seem orthologous to vertebrate NOX4 have been identified in some species but their function and true evolutionary origin and how pervasive they are in the arthropod phylum have never been studied. For a better look of the distribution of NOX4-art genes in arthropod groups we performed tBLASTn searches against the Transcriptome Shotgun Assembly (TSA) sequence database [35] on NCBI website. This enabled us to find NOX4-art orthologs in species of arthropods without sequenced genomes. With NOX4-art searches against the TSA database, 55 sequences belonging to other species of arthropods than those for which we had full genomes were found [see Additional file 2]. Of these, 48 belonged to Hexapoda species, two to Chelicerata and five to Crustacea. Most sequences contained both the ferric reductase and the C-terminal cytoplasmic FAD and NADPH-binding sites. Nevertheless, to make sure the alignments were reliable and enough sites could be used, only sequences longer than 290 amino acids were used in the phylogenetic analysis.

In vertebrates, where these enzymes have been well studied, NOX genes 1 to 4 are regulated by a number of other proteins and the binding to the non-glycosylated integral protein p22-*phox* is essential to their function [6, 21, 26]. For that reason, sequences encoding the putative p22-*phox* accessory protein were also searched in the genomes analyzed using the same approach described for the NOX genes. The search for the p22-*phox* gene in the 95 genomes studied yielded 20 genes in 18 genomes, with only the ascidian, *Ciona intestinalis*, and the cephalochordate, *Branchiostoma floridae*, with two copies [see Additional file 3]. The tBLASTn and Exonerate searches yielded one putative new gene for the species *Capitella teleta* that could only be partially predicted. Only for the two Annelida species (the new, partially predicted, gene from *Capitella teleta* and the gene from *Helobdella robusta*) the polybasic and proline-rich regions (PRR) could not be detected. It is important to highlight that orthologs of p22-*phox* were not found in any arthropod genome.

### Phylogenetic analysis

**Fig. 1:**
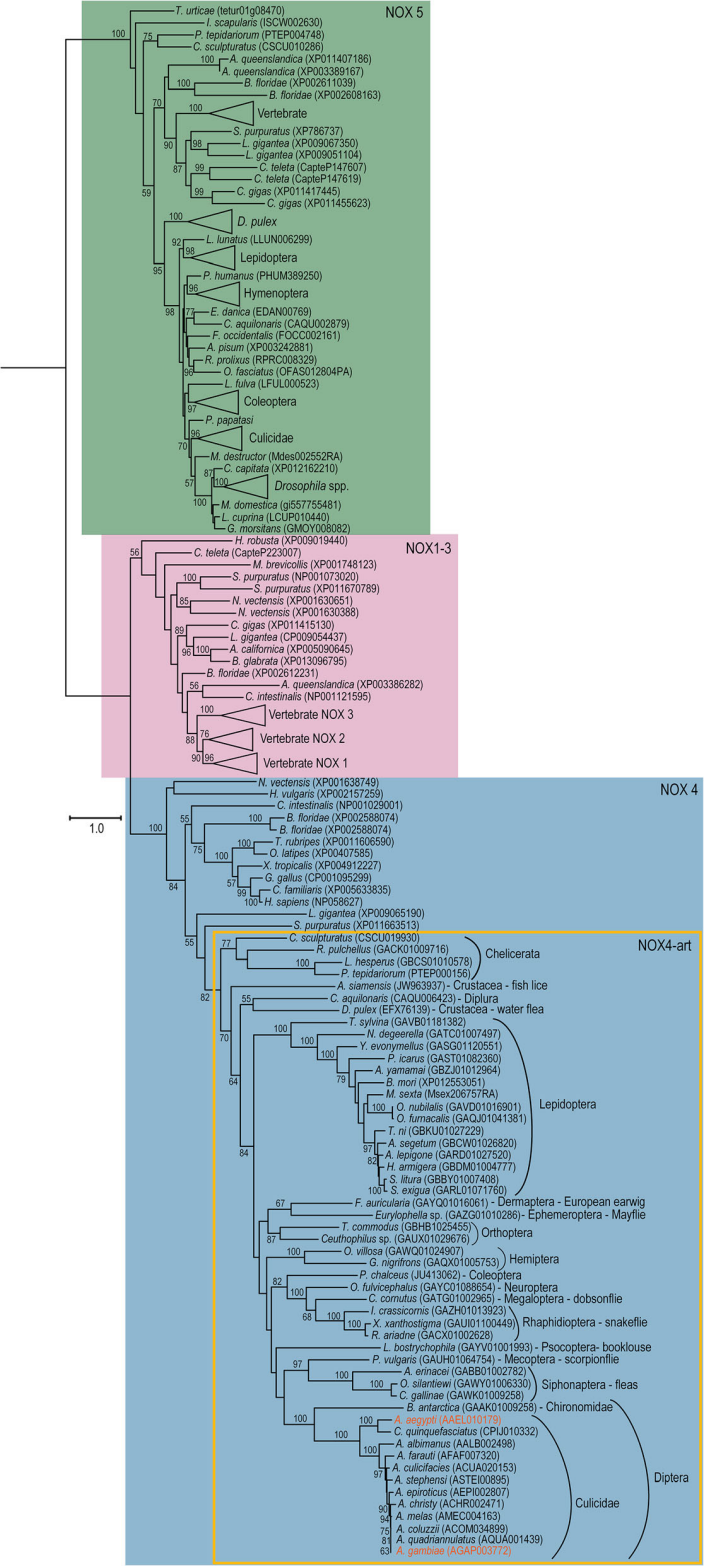
Maximum Likelihood phylogeny of aligned NOX proteins identified in our searches, species names and their accession numbers. Three well-supported clades have been highlighted in different colors: NOX5, NOX1-3 and NOX4/NOX4-art. The yellow square within NOX4 clade depicts the arthropod specific genes (NOX4-art). The *Aedes aegypti* and *Anopheles gambiae* genes have the same sequence as the ones used in Kawahara et al. 2007 [32] and are highlighted in a different color. Numbers on branches are bootstrap support values from 1000 replicates; only numbers above 50% are shown. Scale bar is substitutions per site. The image was created using iTOL [100]

Our phylogenetic analysis using the protein alignment of the ferric reductase and dehydrogenase (FAD and NADPH-binding sites) conserved domains of each sequence retrieved from the 95 metazoan genomes plus arthropod TSA sequences was able to divide the NOXes into four well-supported clades [see Additional file 4]. The DUOX subfamily (92% bootstrap [see Additional file 4]) is the most pervasive with almost all species having at least one gene (exceptions being the Annelida species *Helobdella robusta*, the two cnidarians *Nematostella vectensis* and *Hydra vulgaris* and the Choanoflagellida *Monosiga brevicollis*). These same species also lack a NOX5 gene (clade with 100% bootstrap support [see Additional file 4]). This could be due to genome assembly errors or this gene may have been lost in these lineages. Other Annelida species do have NOX5 and DUOX genes [6] and we did find these genes for the species *Capitella teleta* [see Additional file 1]. However, no NOX5 nor DUOX genes have ever been found in Cnidaria or Choanoflagellida [6, 7]. In agreement with the literature, we did not find a NOX5 gene in the Urochordate species *Ciona intestinalis* [7, 32, 36]. Of the five Arachnids, only in *Metaseiulus occidentalis* no NOX5 was found, suggesting an assembly error in this case. As expected, no NOX5 gene was found in Nematodes [6, 32]. Also, in agreement with Kawahara et al. 2007 NOX1 was only found in vertebrates and NOX3 in mammals and reptiles/birds (Fig. 1) [32]. The NOX1-3 clade also has a high bootstrap value (83%) but the relationships within the clade are not well defined with low bootstrap on most branches (Fig. 1). This could be due to saturation and loss of phylogenetic signal at these deep nodes or, less likely, because most substitutions have occurred within the different lineages sampled and not between them. In vertebrates, NOX1-3 forms a well-supported clade indicating that all three genes have diverged more recently from an ancestral vertebrate NOX2-like gene. Although Zhang et al. 2013 used the terminology NOX1-3 for NOX2 of non-vertebrate metazoans [7], we felt that it was confusing and, for the lack of a better name, we will refer to non-vertebrate NOX2 as NOX2-like. As expected, no NOX2-like gene was found in Ecdysozoa, pointing to a probable loss in their ancestral lineage. NOX4 forms a well-supported clade (100% bootstrap, Fig. 1). The divergence between the NOX1-3 and NOX4 clades probably happened in the ancestral eumetazoan as indicated by the presence of a NOX2-like in the choanoflagelate and Porifera species but of NOX4 only in Cnidaria (Fig. 1, [see also Additional file 1]). Although it was suggested that the sea urchin does not have a NOX4 gene [6], we found one that clusters with good support with other metazoan NOX4. A well-supported clade (82% bootstrap value) nested within the NOX4 genes is comprised only of arthropod genes. This clade includes the NOXm gene found in mosquitoes [32]. With our thorough search of the genomes and TSA database we found that this same gene is present in many other arthropod species. Therefore we suggest it to be renamed as NOX4-art instead of NOXm. The presence and absence of each NOX subfamily and p22-*phox* gene for each animal lineage is summarized in Additional file 1. When more than one sequence of NOX5, NOX2 or NOX4 was found for one particular species these copies seemed to be derived from duplications within the species genome and are therefore paralogs (Fig. 1).

### Synteny and protein domain and motif analysis

For the synteny analysis, only species with complete genomes where NOX4-art was found were analyzed together in the orthoMCL searches. Although many species of *Anopheles* have their genomes sequenced, we chose to use only *Anopheles gambiae* since it is the best annotated and studied species. Within the species studied, synteny analyses revealed conservation of orthologous genes only among species of the same taxonomic group. Within the Culicidae (mosquitoes), NOX4-art genes are flanked by several orthologous groups [see Additional file 5]. Within three of the orthologous groups, paralogous genes were found indicating duplications within a species. Besides putative duplication events, we also found inversions and changes in gene orientation [see Additional file 5]. Taking *Anopheles gambiae* chromosome as reference, the whole syntenic block is inverted in *Aedes aegypti* and, in *Culex quinquefasciatus*, inversions were detected in two pairs of genes, one upstream (CPIJ010328 and CPIJ010329) and one downstream (CPIJ010333 and CPIJ010334) of NOX4-art. In order to detect if this syntenic region was still present in *Drosophila melanogaster*, a species that lacks NOX4/NOX4-art, we used the genes found in this region in mosquitoes as queries for online BLASTp searches against *D. melanogaster*’s genome in GenBank’s nr database. Although some orthologs were found in the fruit fly’s genome, they were not found in the same gene cluster and even belonged to different chromosomes (2L and 3R; [see Additional file 5]). Synteny analysis in both lepidopteran scaffolds revealed two and five pairs of orthologous genes up and downstream of NOX4-art [see Additional file 5].

**Fig. 2:**
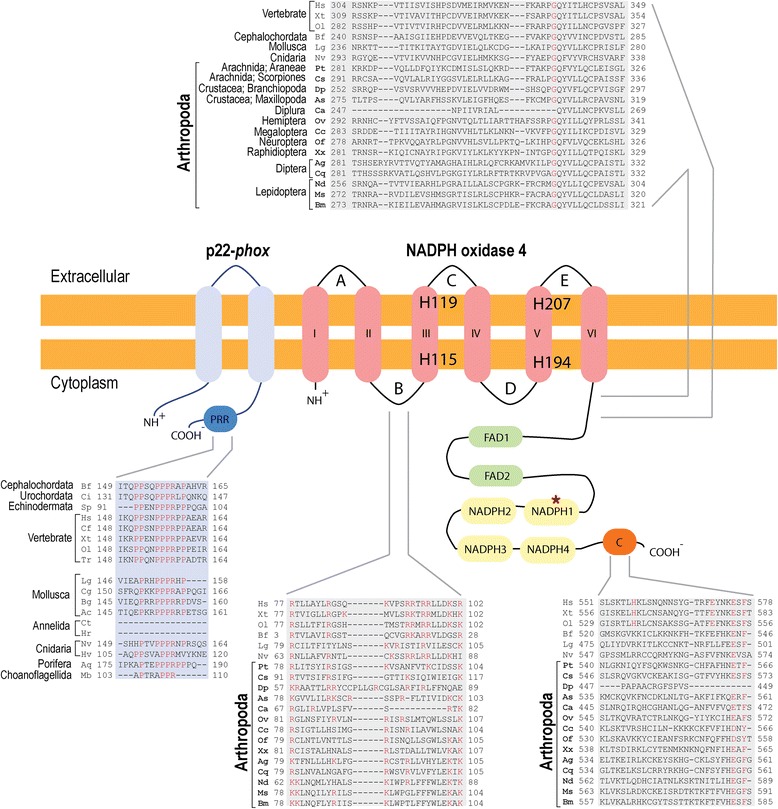
Schematic representation of NOX4/NOX4-art and p22-*phox* proteins with partial regions of the alignment of important loops and segments. The six hydrophobic helixes of the ferric reductase domain are depicted in pink with the four histidine residues. The dehydrogenase domain is colored in *green* and *yellow* (FAD1-2 and NADPH1-4 respectively). The red asterisk in NADPH1 shows where the VXGPFG-motif is located. The C-terminal region, important for the interaction with p22-*phox*, is dark orange. Segments and loops are black with loops identified by capital letters. The protein p22-*phox* with its two transmembrane helixes and proline-rich region (PRR) is illustrated in blue. Partial alignments of important regions are highlighted in blue for p22-*phox* (where no arthropod species are present) and grey for NOX4/NOX4-art. Within the alignments important residues are colored in *red*. Hs - *Homo sapiens*, Xt - *Xenopus tropicalis*, Ol - *Oryzias latipes*, Bf - *Branchiostoma floridae*, Lg - *Lottia gigantea*, Nv - *Nematostella vectensis*, Pt - *Parasteatoda tepidariorum*, Cs - *Centruroides sculpturatus*, Dp - *Daphnia pulex*, As - *Argulus siamensis*, Ca - *Catajapyx aquilonaris*, Ov – *Okanagana villosa*, Cc - *Corydalus cornutus*, Of - *Osmylus fulvicephalus*, Xx - *Xanthostigma xanthostigma*, Ag - *Anopheles gambiae*, Cq - *Culex quinquefasciatus*, Nd - *Nemophora degeerella*, Ms. - *Manduca sexta*, Bm - *Bombyx mori*, Ac - *Aplysia californica*, Aq - *Amphimedon queenslandica*, Bg - *Biomphalaria glabrata,* Cf - *Canis familiaris*, Cg - *Crassostrea gigas*, Ci - *Ciona intestinalis*, Ct - *Capitella teleta*, Hr - *Helobdella robusta*, Hv - *Hydra vulgaris*, Mb - *Monosiga brevicollis*, Sp - *Strongylocentrotus purpuratus*, Tr - *Takifugu rubripes*

For all NOXes, specific structural features have been identified and many key residues and loop sizes were described as important for ROS production and structural stability of the enzymes [32, 37, 38]. Aligning the proteins of the NOX4/NOX4-art clade separately and using the human NOX4 as a guide, we identified previously described conserved amino acid residues, loops and segments. Figure 2 depicts a schematic representation of NOX4 structural features. As expected, all NOX4/NOX4-art proteins had the six transmembrane helixes, two FAD binding and four NADPH binding domains. All four histidine residues that are essential for heme binding and electron transport were present in most proteins. Only in those that did not seem complete, probably due to prediction problems, some important residues could not be found [see Additional file 6]. Loops A, C, D and the segment between NADPH3-4 were highly conserved in the number of residues they contain (Table 1). Apart from loop C, loops A and D and the segment between NADPH3-4 were also conserved in size among all NOX1-4 proteins, indicating that the size of the loops might be important for protein function [32]. In NOX4/NOX4-art, loop E varies in size among the different taxonomic groups (Table 1). Kawahara et al. 2007, identified this segment as being longer in all NOX4 genes when compared to NOX1-3 [32]. Nevertheless, in our study, with a higher number of taxa of different taxonomic groups, we can see that in some cases the loop is long but in other cases it is as small as in NOX1-3 (41-44 residues in [32], Table 1). One differentiating portion of NOX4/NOX4-art is loop C, that seems to be conserved in size in our analysis (Table 1) but longer in NOX1-2 (37-41 residues) and NOX3 (38 residues) [32]. In loop E, deletion of the THPPGC motif or mutation of the histidine (in position 222) and cysteine residues (in positions 226 and/or 270) switches hydrogen peroxide to superoxide production in NOX4 [39]. In arthropods, although the motif is different, the histidine and cysteine residues are present in most species [see Additional file 6]. Loop B (and arginine and lysine residues within), segment TM6-FAD (and a glycine residue within), the VXGPFG-motif (within NADPH1) and the extreme C-terminal regions (and glutamate and phenylalanine residues within) were identified as being important for NOX activity [32, 37, 38] (Fig. 2). All species have a polybasic-rich region in loop B, characterized by arginine and lysine residues [see Additional file 6]. In human NOX4, these residues are functionally important and bind to the C-terminal region of the DH (FAD/NADPH-binding) domain, providing proximity for the transmembrane heme-binding domain to interact with it [40]. Although many arginine residues are present in loop B of arthropod’s NOX4-art genes, the RRXRR motif characteristic of vertebrates [37] is not present (Fig. 2 [see also Additional file 6]). In segment TM6-FAD, most NOX sequences have the glycine residue near position 336. In vertebrate NOX2, this residue and an arginine near position 80 in loop B, also present in most of our sequences (a lysine substitutes the arginine in Diptera), are thought to participate in the binding of p22-*phox* [41]. The expected glutamate (position 575) and phenylalanine (position 577) residues are present in the C-terminal region of all sequences but the histidine (position 557) and a second glutamate (position 571) residue are absent in most arthropod sequences (Fig. 4 [see also Additional file 6]). Since in the NOX4/p22-*phox* complex these residues are necessary for the constitutive production of ROS [37], their absence in NOX4-art sequences indicates that these genes are either not constitutively active or that the interaction of loop B and the C-terminal region might be different from that of vertebrate NOX4. All sequences have the VXGPFG-motif within NADPH1 binding site.

**Table 1:** Number of amino acids present in loops and segments joining transmembrane (TM) regions and canonical DH domains in NOX4/NOX4-art of the different taxonomic groups analyzed. # - Number of species in each taxonomic group

Group	#	loop A	loop B	loop C	loop D	loop E	TM6	-	FAD1-2	-	NADPH1	-	NADPH2	-	NADPH3	-	NADPH4	C-terminus
Cnidaria	2	16	26-27	25	13	41-73		46		49-69		19-20		49		20		28
Arachnida	5	16	27	25	13	45-76		46-51		43-57		15-16		51-52		20		27-29
Crustacea	6	16	12-33	15/25	13	12-40		27-46		36-58		7-11		48-52		20		27
Hexapoda^a^	62	16	21	25	13	11-69		46-52		36-52^b^		14-17		51-52^c^		20		27-30
Echinodermata	1	16	25	25	13	76		204		45		19		49		20		28
Urochordata	1	16	26	25	13	135		47		109		18		48		22		27
Cephalochordata	1	0	26	25	13	80-82		46		52-75		16		48		20		27
Vertebrates	6	16	26	25	13	48-75		46		48		15-16		48-49		20		28

^a^At least in 80% of the species analyzed; ^b^varies among orders; ^c^much bigger in Lepidoptera (median = 102.5)

### Silencing of NOX4-art

**Fig. 3:**
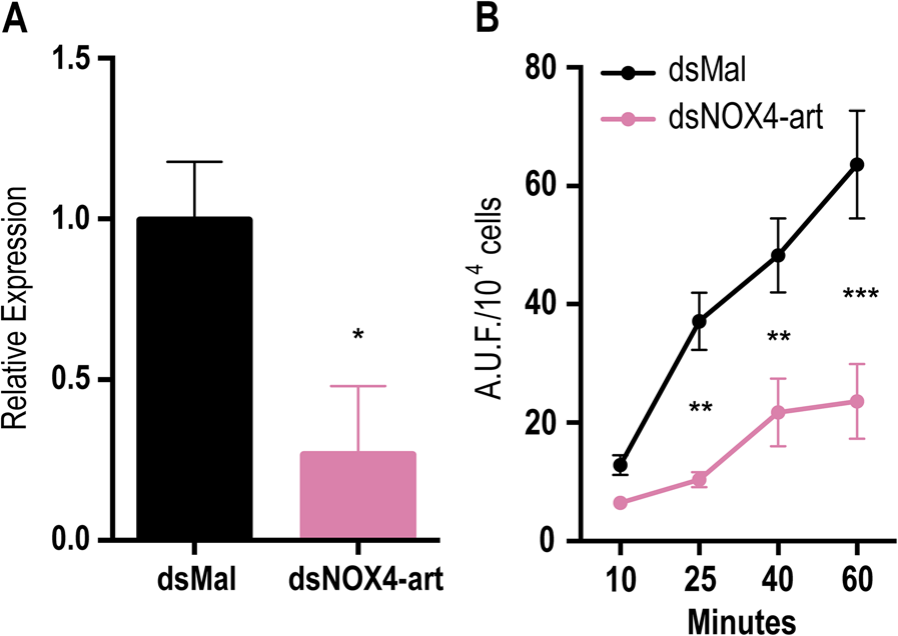
The silencing of NOX4-art by dsRNA decreases hydrogen peroxide production in Aag-2 cells. **a** qPCR assays were performed with Aag-2 cells 4 days after transfection with dsRNA. Error bars indicate the standard error of the mean. *p < 0.005 (Student’s t-test). **b** Hydrogen peroxide production by Aag-2 cells was inferred by Amplex Red assay. Results are pools of 2 independent experiments. Error bars indicate the standard error of the mean. **p < 0.01 and **p < 0.0001 (One-way ANOVA, Sidak’s test)

As p22-*phox* has been shown to be essential for enzymatic activity in the NOX4 proteins studied so far, the presence of the NOX4-art genes in arthropods, together with the lack of p22-*phox* could be interpreted as indicative of absence of catalytic activity. In order to test for the ROS producing activity of NOX4-art, we used dsRNA-mediated silencing of the NOX4-art gene in *Aedes aegypti* embryonic cells (Aag-2). NOX4-art silencing resulted in a significant decrease in hydrogen peroxide production (Fig. 3a and b), clearly demonstrating that the protein coded by the NOX4-art gene is catalytically active, possibly by direct production of hydrogen peroxide, as shown for vertebrate NOX4 and DUOX1-2 [2].

## Discussion

In this study, we evaluated the diversity of NOXes in 70 arthropod genomes and investigated the origin and function of a NOX gene previously found only in the mosquitoes *Anopheles gambiae* and *Aedes aegypti*, here shown to be an ancestral trait of the arthropod lineage. The annotation of the complete NOX repertoire in arthropods allowed us to better understand gene divergence and the importance of deletion events in the evolution of this essential gene family. Combining the phylogenetic, domain and residue analysis and laboratory work, our data would suggest that: 1) Indeed it seems that a NOX2-like gene was lost in the ancestral lineage leading to Ecdysozoa; 2) As suggested before, NOX5 and DUOX are present in all arthropod lineages; 3) NOX4-art evolved from a NOX4-like ancestor; 4) NOX4-art is functional and, although no p22-*phox* was observed in arthropods, there was no indication of neo-functionalization as this gene still produces hydrogen peroxide; 5) Although functional and present in the genomes of many species, NOX4-art was lost in a number of arthropod lineages. Public domain database’s automatic blast similarity searches have already indicated that a NOX4-like gene is present in some arthropod species. Nevertheless, to our knowledge, this is the first work to show that NOX4-art is an arthropod specific new gene that originates from a NOX4-like ancestor in a phylogenetic framework. We show here the evolutionary origin of this new gene and how pervasive it is in arthropod phylogeny although it has also been lost in many species.

### Evolution of NOX genes

A previous study that analyzed the NOX family in a larger context found that this ROS generating family is monophyletic and clusters together with other ferric reductase genes. It was proposed that the shift in function from metalloreductase to ROS production took place in the ancestral gene of early eukaryotes [7]. In agreement with other phylogenetic studies, it was also suggested that since NOX2-like is present in choanoflagellates and that sea anemones have both NOX2-like and NOX4, the split between these two clades happened in the ancestral metazoan. We have not found NOX4 in the sponge genome, indicating that the NOX4 gene might have actually emerged later at the time of the cnidarian-bilaterian divergence or may have been lost in this sponge species. These two clades arose from EF-hand containing genes (Ca^2+^-dependent NOXes) and the ancestral NOX2-like gene later lost these domains [7]. This would suggest that both NOX5 and DUOX were later lost in choanoflagellates and cnidarians. Gene loss seems to be common in the NOX family evolution [6]. NOX5, for example, was also lost in the phylum Nematoda [6, 32] and in the mammalian order Rodentia [6]. Our results also corroborate the loss of NOX2-like in the Ecdysozoa lineage [6, 32]. In fact, Ecdysozoans seem to have suffered extensive gene losses in many gene families throughout their evolution [42].

Gene duplication followed by subfunctionalization or neofunctionalization has been proposed to facilitate the evolution of new gene functions [43–45]. Nevertheless, the evolutionary impact of lineage-specific gene losses has never gained much attention. If no paralogs are present, a gene function that is exclusively associated with a certain gene may disappear if that gene is lost. This outcome has been thought of as detrimental to the species, rendering it less adaptable to the changing environment. However, recently, with new methodological and technological advances (great number of genomes sequenced), more evidence of the pervasiveness of gene loss has been gathered [46]. It is now contemplated that gene loss can be neutral or even adaptive and thus relevant for species evolution (for a review see [42]). Castro et al. 2014, for example, have found that gene loss, related to the gastric function gene kit, correlates with the evolution of different stomach types in vertebrates, which might be associated with their diet [47]. Also, the absence of some urea cycle and essential amino acid synthesis enzymes in the hematophagous *Rhodnius prolixus* was speculated to be due to relaxation of these pathways in an amino acid rich diet [48].

The evidence for gene loss is negative and can pass unnoticed or not be considered due to uncertainties in the completion or assembly of sequenced genomes. Therefore, the impact of gene loss in the evolution and function of surviving paralogs is not well investigated. It is easier to recognize gene duplication and the appearance of a new gene function as adaptive. In the evolution of the NOX family, gene duplication followed by neofunctionalization seems to have happened very early since both the ability to produce superoxide and hydrogen peroxide were present in the ancestral calcium binding enzymes (NOX5 and DUOX, respectively) [7]. In addition, further evidence of subfunctionalization has recently been gathered in vertebrates. Vertebrate NOX2 seems to be expressed mostly in phagocytes whilst NOX1, NOX3 and NOX4 have other specific functions and patterns of subcellular localization and tissue distribution [2, 19, 49]. Still, the major characteristic of this family of enzymes seems to be evolution by gene loss.

### Gene loss and NOX4-art evolution

**Fig. 4:**
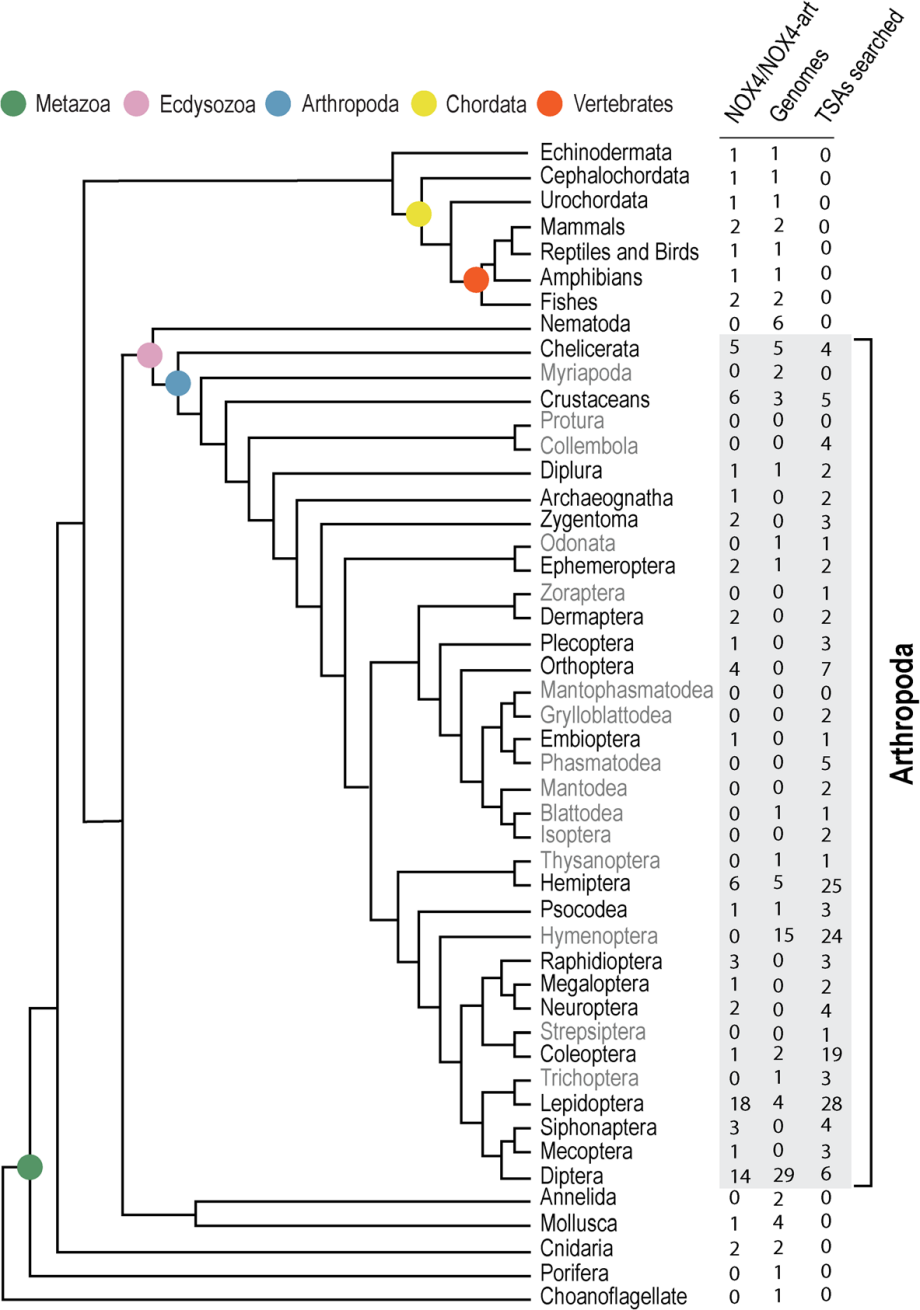
Current taxonomic tree of metazoans and Choanoflagellida with emphasis on arthropod lineages. Phylogenetic relationships between the taxonomic groups adapted from Misof et al. and Dunn et al. [57, 101]. Number of NOX4/NOX4-art genes and the total number of species analyzed here (among genomes and TSA experiments searched) are represented in the columns. Names of taxonomic arthropod orders in grey had no NOX4-art. The grey square delimits the phylum Arthropoda

Our more extensive search shows that, as with NOX2-like, the NOX4/NOX4-art gene is present in more animal groups than previously thought [32] but was also lost in many lineages throughout metazoan evolution. This gene seems to have been lost in Nematoda and Annelida and, of the four molluscan genomes searched, it was found in only one. In these phyla, however, only a small number of genomes were searched and no transcriptomes. In arthropods, where a larger sampling scheme was performed, we show here that NOX4-art was lost many times during evolution (Fig. 4). Although present in Chelicerates, Crustaceans and Hexapods, it was not found in the two Myriapoda genomes searched (Fig. 4).

In Hexapods, sometimes the gene seems to have been lost in the ancestral lineages of whole orders (in Hymenoptera, for example) but this has happened mostly in different species within different taxonomic groups (clades). It is important to differentiate species where only transcriptomes were analyzed from those where complete genome assemblies are present (Fig. 4) since the absence from a transcriptome may only mean the gene was not being transcribed at that particular time. Nevertheless, even if we take into consideration that the gene could not be found in the genomes of a few species due to assembly errors, we would still see that it is absent from a variety of Hexapod species. In Diptera, for example, NOX4-art is found in mosquitoes but not in the extensively studied genomes of the different *Drosophila* species. This pattern of evolution certainly fits the “patchy ortholog” design that is commonly found in ancient gene families [42].

The pervasiveness of gene loss begs the question of how many genes are actually indispensable in any given genome. In *Drosophila melanogaster*, it has been suggested that around 85% of its genes are dispensable [50]. Reductive evolution in parasitic or symbiotic species is not a new concept but for other species gene loss has usually been linked to reduction in fitness or has been associated to adaptiveness to specific environments such as the loss of genes related to vision in cave dwelling animals, for example [51–53]. Another possibility is the presence of other genes such as paralogs, analogs or even whole different pathways that serve the same or very similar functions and therefore the loss of a specific gene does not mean loss of function (mutation robustness). Since NOX4-art produces the same byproduct (hydrogen peroxide) as DUOX enzymes, it is plausible that this redundancy might be the reason why it was lost in some lineages. However, this does not explain why in other lineages the gene is present and functional. If DUOX and NOX4-art roles and expression patterns are exactly the same, one questions if a dosage balance problem could not have arisen.

Comparative analyses of gene losses according to gene ontology (GO) categories have shown that the differences in dispensability observed between different genes might not be stochastic. GOs related to signal transduction, one of the main functions attributed to NOX4 enzymes [54], and other functions that are more sensitive to dosage imbalance are more prone to be lost [42]. Other GO categories of more ancient biochemical processes such as protein modification and immune response, among others, were also deemed more prone to gene loss in different organisms. All of these are functions in which the production of ROS by NOX enzymes seems to be involved in. This family of enzymes is certainly a success story that has arisen early in the evolution of multicellular life and thus their participation in ancient biochemical processes and patterns of gene gain and loss are expected [2, 55, 56]. However, it is also possible that the loss of a gene function altogether might not have a detrimental or adaptive effect on a species and, in fact, can be neutral [46]. Neutral or nearly neutral gene losses can be fixed in a species through genetic drift and, in ancient gene families such as NOXes, the effect on a phylogenetic framework can be seen as the patchy distribution of orthologs observed [42, 46].

Although it would have been desirable to be sure whether incomplete genome coverage and/or assembly or true gene loss was the cause behind the apparent absence of NOX4/NOX4-art in many species, a synteny analysis to examine the genomic *loci* was simply not possible for most groups. A strong degree of synteny was only possible to be seen in closely related species probably due to the great divergence among the many species studied (diverse metazoan groups). Even though hexapods are a more closely related group, the lack of synteny found among the different orders may be due to their ancient radiation (479 million years ago [57]) and to the great amount of genome rearrangements that might have occurred. Indeed, much faster rates of chromosomal rearrangements have been found for *Drosophila* species when compared to other eukaryotes [58, 59]. In addition, for this genus, high rearrangement rates were also found for co-expressed genes within short intergene sequences [60]. Extensive genome re-arrangements among mosquito (*Anopheles gambiae*) and fly lineages have also been found [61]. Therefore, it is not surprising then that in our study we have not found synteny between the three mosquitoes analyzed and the *Drosophila melanogaster* genome. Furthermore, the lepidopteran orthologous groups belong to completely different ones than those of the mosquitoes, indicating that there is no special region on the genome for the NOX4-art gene. It seems that it has changed places in the genomes as much as any other old gene and does not seem to belong to any specific expression or otherwise constrained synteny block [58, 59].

### NOX4-art function and activation

The arthropod NOX4-art clade includes the mosquito gene previously described as NOXm [32], indicating that this gene is more widely distributed within the phylum and is not related to hematophagy as previously thought. Arthropod NOX4-art formed a well-supported distinct group closely related to NOX4 (Fig. 1). Indeed, our functional analysis indicates that this gene still produces hydrogen peroxide as other NOX4 genes.

Although NOX4-art is closely related to NOX4, its expression and stability might be different. No p22-*phox* was found in any arthropod indicating that a different protein or mode of action might be responsible for NOX4-art activation. When the expression of p22-*phox* is inhibited, it greatly diminishes the production of ROS by NOX4. Nevertheless, neither truncated nor mutated forms of p22-*phox* that disrupted the PRR had any effect on ROS production on vertebrate NOX4 genes [21]. Since this region is the most characteristic of p22-*phox* in most metazoans, it might be possible that another protein that we could not find with our searches using these proteins as queries could be acting as a NOX4-art activator. It has been proposed that NOX4/p22-*phox* complex is structurally different from NOX1-3 with loop D being the most important feature for the formation of the complex. NOX4 chimeras with NOX2 loop D sequences did not translocate to the cell surface nor produced ROS [38]. Nevertheless, a specific motif or residue has not been attributed to the formation of this complex and a better understanding of the interaction of these two proteins is still needed.

Motifs and residues in loop B and DH domain are essential for ROS production since their interaction approximates the heme, FAD and NADPH binding sites, facilitating the transfer of electrons. Although the characteristic mammalian RRXRR loop B motif is not present in NOX4-art, it is also absent in other metazoan NOX4. This motif has been determined as important for ROS production in mammalian NOX4 but mostly because of the first arginine residue [37]. Arginine residues are present in most NOX4-art loop B sequences although not in that specific motif. It seems plausible that although different from the vertebrate motif other positively charged motifs might be responsible for the interaction of the DH domain and loop B and, therefore, NOX4-art activity. The C-terminal region of NOX4 is also important for catalytic activity. Substitution of the amino acids histidine and glutamate or changes in the size of this region substantially decrease ROS production in vertebrates. These changes alter the distance between FAD and NADPH binding regions and hinder the proper folding of the DH domain [37]. The glutamate residue seems to be present in most NOX4-art sequences and the number of residues in the C-terminal region does not vary much among arthropod groups being well within the range found for vertebrates (Table 1). The position of the histidine residue varies in NOX4-art; which might not be a problem since in loop B the motifs are different from vertebrates and the relative position of these two regions, that have to interact for proper ROS production, may be different for this gene when compared to vertebrate NOX4. Indeed, although there are many differences in important functional residue positions and motifs between vertebrate NOX4, other metazoan NOX4 and NOX4-art, the silencing of *Aedes aegypti* NOX4-art shows us that this gene is functional and probably produces hydrogen peroxide as expected for a NOX4 gene.

When VectorBase (www.vectorbase.org) expression maps are searched for *Anopheles gambiae* and *Aedes aegypti* NOX4-art genes (AGAP003772 and AAEL010179, respectively), we find differential expression during embryonic development [62, 63] and between tissues [64, 65] and increased expression after a blood meal [66, 67]. In *Aedes aegypti*, infection with *Micrococcus luteus* or *Wolbachia* (wMel strain) also increases NOX4-art expression [68, 69]. Thus, it seems that NOX4-art, in mosquitoes at least, might be linked to a number of physiological functions.

## Conclusions

The increasing number of genomes available can significantly contribute to the study of gene family evolution. The NOXes are an intriguing gene family that is responsible for important cellular processes such as cell signaling, transcriptional regulation, stress response and gut homeostasis and defense. Genes from this family are present in all eukaryotic groups analyzed until now suggesting that the common ancestor of NOX genes emerged at an early stage in the evolution of eukaryotes.

We have found that the NOXm (now renamed NOX4-art) gene described for mosquitoes is actually widespread within arthropods. This gene is potentially functional where it is present and probably produces ROS in the form of hydrogen peroxide. Important insect specific functions were described for NOXes such as cuticular hardening [70, 71], wound healing [72, 73], smooth muscle contraction [74] and gut immunity [34, 75–77]. NOX4-art could have an arthropod specific function as well and therefore be an interesting target for new vector and pest control strategies. In addition, non-mammalian experimental systems are advantageous due to the smaller number of genes present which helps to elucidate questions regarding their expression in different developmental stages and/or tissues.

The arthropod NOX4-art gene is also an interesting example of evolution by gene loss. Within the phylum Arthropoda it has a ‘patchy’ distribution with some species possessing the gene while others do not. It is difficult to determine if the loss of the NOX4-art gene in different lineages is due to the presence of another enzyme with similar function (DUOX) or simply a neutral pattern of evolution. Dosage imbalance does not seem to be a problem where NOX4-art is present, although subfunctionalization has not been discarded. One thing is certain though; the study of gene loss in gene family evolution should receive more attention, as it could be an important source of information about evolutionary patterns and processes.

## Methods

### Gene search

To search for NOX genes in the 95 genomes analyzed, the ferric reductase transmembrane component domain (HMM profile PF01794) [78] was used as query in HMMsearch [79] using the FAT pipeline [80]. All proteins with significant E-value (<0.001) were retrieved and used as queries on BLASTp searches [81] against the manually curated Uniprot/SwissProt protein database [82] also using FAT. To make sure that all genes belonging to the NOX gene family were found in arthropods and other genomes, tBLASTn and Exonerate (protein2genome mode) [83] searches against the scaffolds of the whole genome databases were also performed. This makes it possible to find genes that might not have been predicted before and therefore could not be retrieved with the HMMsearch step. These searches were performed with the already predicted NOX genes of other closely related species as queries. Redundancy was eliminated with the software CD-Hit [84]. When new peptides were found they were predicted with GeneWise [85], using the closest BLASTp search homolog to predict a full-length protein sequence, when possible. The results were visualized and further edited, when needed, in the genome browser Artemis [86].

We also performed tBLASTn searches against the Transcriptome Shotgun Assembly (TSA) sequence database [35] at the NCBI website using *Anopheles gambiae* (AGAP003772), *Bombyx mori* (XP_012553051), *Catajapyx aquilonaris* (CAQU006423) and *Daphnia pulex* (EFX76139) NOX4-art sequences. These sequences were the ones that seemed better annotated and spanned a wide taxonomic diversity from our previous search. The mRNA best hits were saved in FASTA format and redundancy was removed with CD-Hit [84] with a cut off of 100%. Artemis [86] was used to extract the coding sequence and the respective peptide sequence for each hit. Conserved domain composition was confirmed by searches against the Protein Family Database (Pfam) and Conserved Domain Database (CDD) [78, 87].

Sequences encoding putative p22-*phox* accessory protein were also searched in the genomes analyzed using the same approach described for the NOX genes above. The cytochrome b558 alpha-subunit domain (HMM profile PF05038) was used as query for HMMsearch. Proteins with significant E-values (<0.001) were retrieved and used as queries on BLASTp searches against Uniprot/SwissProt. Again, whenever no hits were found tBLASTn and Exonerate searches against the scaffolds of the whole genome databases were also performed. This search ensured that we could find genes that were not automatically predicted.

The sequences of all peptides used in the phylogenetic analysis and their accession numbers are available for download in FASTA format as supplementary material [see Additional file 7].

### Phylogenetic analysis

Amino acid sequences of the proteins retrieved by our searches of genomes and the TSA database were aligned locally with PASTA [88] using the JTT + G20 model and other default parameters. The alignments were visualized and converted to Phylip format using the software SeaView [89]. The same program was used to trim the sequences leaving only the region containing the ferric reductase and the dehydrogenase (FAD1-2 and NADPH1-4) domains that are common to all NOX/DUOX genes. This way, the peroxidase domain found only in DUOX genes and the calcium binding domains present in both DUOX and NOX5 genes were eliminated from the alignment. This trimmed version of the alignment was then used to construct a phylogenetic tree using the maximum likelihood method with RAxML [90] on CIPRES web server [91]. The amino acid Jones Taylor Thornton scoring matrix was used [92] and bootstrap analysis with 1000 replicates was performed to infer branch support.

### Synteny and protein domain and motif analysis

For all genes CD-Search Batch (CDD v3.14 database) [93] and BLAST searches on Pfam 28.0 [78] were used to infer conserved sites and to confirm the protein domain structure. TMHMM [94] available on the web was also used to infer the presence and position of the transmembrane helixes. To investigate NOX4-art neighborhood microsynteny we used genomes of the species *Aedes aegypti*, *Culex quinquefasciatus*, *Anopheles gambiae*, *Bombyx mori*, *Manduca sexta*, *Catajapyx aquilonaris*, *Centruroides sculpturatus*, *Daphnia pulex*, *Parasteatoda tepidariorum* and *Tetranychus urticae*. We extracted all translated coding sequences present from each side of NOX4-art genes in their scaffolds/chromosomes. OrthoMCL [95] was used to group putative orthologues with an E-value lower than 10E-5. For the motif analysis, the amino acid sequences retrieved by the FAT pipeline were aligned with ClustalW [96] within BioEdit software [97]. Vertebrate NOX4 was used as a guide for NOX4-art analysis. Based on Kawahara et al. 2007 loop sizes, canonical regions and conserved amino acid residues required for ROS production were searched in the alignment and were identified manually [32].

### NOX4-art silencing

*Aedes aegypti* Aag-2 cells were cultivated in 25 cm^2^ plastic flasks in Schneider’s *Drosophila* medium supplemented with 10% fetal bovine serum until 100% confluence and then maintained at 28 °C. Cells were transfected using the cell line Nucleofector kit V according to the manufacturer’s instructions (Amaxa Biosystems, Köln, Germany). Briefly, cells (1 × 10^6^) were centrifuged and carefully resuspended in 3 μg of dsRNA (dsMAL and dsNOX4-art) and 100 μL of transfection reagent (82 μL cell line plus 18 μL supplement). The unrelated dsRNA, dsMal, specific of *Escherichia coli* MalE gene (Gene ID: 948538), was used as a control for the off-target effects of dsRNA. The cell/dsRNA suspensions were transferred into a Lonza certified cuvette and transfected in the Nucleofector I Device (Lonza, USA) with the program G-030. Immediately after transfection, 2.4 mL of Schneider’s supplemented media was gently added to the cuvette and the cells were seeded into a 96 well plate at a density of 4 × 10^4^ cells per well. After that, cells were incubated at 28 °C in an air incubator until analysis.

### RNA extraction and qPCR

Total RNA was extracted from Aag-2 cells (4 x 10^4^) using TRIzol (Invitrogen) according to the manufacturer’s protocol. One microgram of RNA was treated with RNasefree DNase I (Fermentas International Inc., Burlington, Canada). The treated RNA was used to synthesize the cDNA with the High Capacity cDNA reverse transcription kit (Applied Biosystems, Foster City, CA). qPCR was performed on a StepOnePlus qPCR system (Applied Biosystems) using the Power SYBR Green PCR master mix (Applied Biosystems). The comparative *Ct* method [98] was used to compare gene expression levels. The *Aedes aegypti* ribosomal protein 49 gene (Rp49) was used as an endogenous control, based on previous data [99]. The primer pairs used for the amplification of cDNA fragments for both conventional and qPCR were: *NOX4-art*: forward 5-TTG TGT TCG CAC ATC CAA CT-3 and reverse 5-GGT CCA ACG AAA AAT ATC CAA A-3; *Rp49*: forward, 5-TGT CGG TGT AAC TGG CAT GT-3 and reverse, 5-TCG GCC AAC AAA AGT ACA CA-3. Statistical analysis was performed with Graphpad Prism software with Student’s t-test.

### ROS measurement

Hydrogen peroxide production by Aag-2 cells was measured by monitoring resorufin fluorescence due to the oxidation of Amplex Red (Invitrogen, USA). Cells (4 × 10^4^ cells/well) were incubated at 28 °C for 4 days after transfection with dsRNA and then, assayed in 50 μM Amplex Red (Invitrogen, USA), 40 units of horseradish peroxidase (Sigma, USA) and 25 units of superoxide dismutase (Sigma, USA) in Schneider’s *Drosophila* medium, for a final volume of 200 μL. Immediately after reagent mixture, the endpoints of Amplex Red oxidation were recorded at room temperature using a Spectra Max spectrofluorimeter (Varian, USA), operating at excitation and emission wavelengths of 530 nm and 590 nm. Statistical analysis was performed with Graphpad Prism software with One-way ANOVA, Sidak’s test.

## Additional files

Additional file 1: Table with a list of the 95 species with complete genomes analyzed, number of proteins found for each NOX gene, their genome source and version. (XLSX 18 kb)

Additional file 2: Table with a list of the species where NOX4-art genes could be found on NCBI’s TSA database. Their TSA identification number and amino acid length. (XLSX 12 kb)

Additional file 3: Table with the list of species where p22-*phox* was found, their gene identification, amino acid length and composition of the polybasic and proline rich region. (XLSX 13 kb)

Additional file 4: Maximum Likelihood phylogeny of aligned NOX and DUOX proteins identified in this study. Numbers on branches are bootstrap support values from 1000 replicates; only numbers above 60% are shown. The image was created using iTOL [100]. (TIFF 7473 kb)

Additional file 5: Micro-synteny around NOX4-art gene (orange arrow) in Diptera and Lepidoptera. Orthologous genes are represented by grey arrows, paralogs by blue arrows and genes where no orthologous or paralogous relationship could be determined within the genomes are depicted in grey-wired arrows. A) Complete chromosomes 2 and 3 of *Drosophila melanogaster* and scaffold/chromosome regions, where NOX4-art was found, in *Anopheles gambiae*, *Culex quinquefasciatus* and *Aedes aegypti*. B) Scaffold regions, where NOX4-art was found, in *Bombyx mori* and *Manduca sexta*. Estimated divergence between *Anoheles gambiae* and other diptera species and the two lepidopteran species are given in million years (MY) [57]. (TIFF 1688 kb)

Additional file 6: Alignment of NOX4/NOX4-art genes organized by taxonomic group. Important regions and residues are highlighted in different colors: green – transmembrane regions; black – heme binding histidines; grey – loops between transmembranes; cyan – different important residues; magenta – FAD binding regions; orange – NADPH binding regions; yellow – C-terminal region. (PDF 3860 kb)

Additional file 7: FASTA file with the peptides used in the phylogenetic analysis and their NCBI accession numbers. (TXT 287 kb)
