# Erratum to: Evolutionary origin and function of NOX4- art, an arthropod specific NADPH oxidase

**DOI:** 10.1186/s12862-017-1041-9

**Published:** 2017-08-21

**Authors:** Ana Caroline Paiva Gandara, André Torres, Ana Cristina Bahia, Pedro L. Oliveira, Renata Schama

**Affiliations:** 1 Instituto de Bioquímica Médica Leopoldo de Meis, Universidade Federal do Rio de Janeiro, Rio de Janeiro, Brazil; 2 Laboratório de Biologia Computacional e Sistemas, Instituto Oswaldo Cruz, Fiocruz, Rio de Janeiro, Brazil; 3 Instituto de Biofísica, Universidade Federal do Rio de Janeiro, Rio de Janeiro, Brazil; 4 Instituto Nacional de Ciência e Tecnologia em Entomologia Molecular – INCT-EM, Rio de Janeiro, Brazil

## Erratum

After publication of the original article [1], the authors noticed the following errors in the Methods section of the manuscript:

1. The dsRNA synthesis had not been included.
2. A portion of the primer sequences for NOX4-art in “RNA extraction and qPCR were incorrect.

Corrections to these errors follow below:

1. The authors intended the below dsRNA synthesis section to be included between the **Synteny and protein domain and motif analysis** and **NOX4-art silencing section.**

**dsRNA synthesis**

Double stranded RNAs for MAL (dsMAL) and NOX4-art (dsNOX4-art) were produced from PCR-amplified fragments using the HiScribe T7 In Vitro Translation Kit (New England Biolabs). Amplicons for dsMAL were produced using plasmid templates and for dsNOX4-art by reverse transcriptase PCR (RT-PCR) products, from sugar-fed female cDNA. Two rounds of PCR were performed to amplify NOX4-art. The first PCR round was performed with primers containing a short adaptor sequence at the 5′ end (tggcgcccctagatg). The primers used for the first round of PCR were NOX4-artFwd 5′ tggcgcccctagatgAGTGGCACCCGTTTACAGTC 3′ and NOX4-artRev 5′ tggcgcccctagatgTTTGGGACCACAACTGAACA 3′. The PCR cycles utilized were 95 °C for 3 min, 35 cycles of 95 °C for 30 s, 57 °C for 45 s and 72 °C for 45 s followed by 72 °C for 7 min. Two microliters of the first PCR were used in the second PCR reaction. The second round of PCR was utilized to insert the bacteriophage T7 DNA-dependent RNA polymerase promoter to the DNA templates. The second round of PCR utilized the same conditions of the first reaction. The second round PCR primer, which has the T7 and the adaptador sequences, was 5′ ccgTAATACGACTCACTATAGGtggcgcccctagatg 3′. We amplified the unrelated maltose transporter subunit gene of *Escherichia coli* (Gene ID: 948,538) from the Litmus 28i-mal plasmid (New England Biolabs) with a single primer (T7, 5′-TAATACGACTCACTATAGGG-3′) specific for the T7 promoter sequence that is on both sides of the MalE sequence [2]. dsNOX4-art or dsMAL (69 nL of 3 μg/μL) were diluted in distilled water.

2. The authors intend that the bolded primer sequences in the below extract should replace what is in the original article (references cited are also present in the main text):

**RNA extraction and qPCR**

Total RNA was extracted from Aag-2 cells (4 × 10^4^) using TRIzol (Invitrogen) according to the manufacturer’s protocol. One microgram of RNA was treated with RNasefree DNase I (Fermentas International Inc., Burlington, Canada). The treated RNA was used to synthesize the cDNA with the High Capacity cDNA reverse transcription kit (Applied Biosystems, Foster City, CA). qPCR was performed on a StepOnePlus qPCR system (Applied Biosystems) using the Power SYBR Green PCR master mix (Applied Biosystems). The comparative *Ct* method [3] was used to compare gene expression levels. The *Aedes aegypti* ribosomal protein 49 gene (Rp49) was used as an endogenous control, based on previous data [4]. The primer pairs used for the amplification of cDNA fragments for both conventional and qPCR were: *NOX4-art*: forward 5-**GGACAGGCGAAAAGTATCCA**-3 and reverse 5-**GACTGTAAACGGGTGCCACT**-3; *Rp49*: forward, 5-TGTCGGTGTAACTGGCATGT-3 and reverse, 5-TCGGCCAACAAAAGTACACA-3. Statistical analysis was performed with Graphpad Prism software with Student’s t-test.

- 5-**GGACAGGCGAAAAGTATCCA**-3 replaces 5-TTG TGT TCG CAC ATC CAA CT-3 in the original article.
- 5-**GACTGTAAACGGGTGCCACT**-3 replaces 5-GGT CCA ACG AAA AAT ATC CAA A-3 in the original article.
