# Supplementary figures and images for: Evolutionary origin and function of NOX4-art, an arthropod specific NADPH oxidase
Source: BMC Evol Biol. 2017 Mar 29;17:92. doi: 10.1186/s12862-017-0940-0 (PMC5372347; doi:10.1186/s12862-017-0940-0)

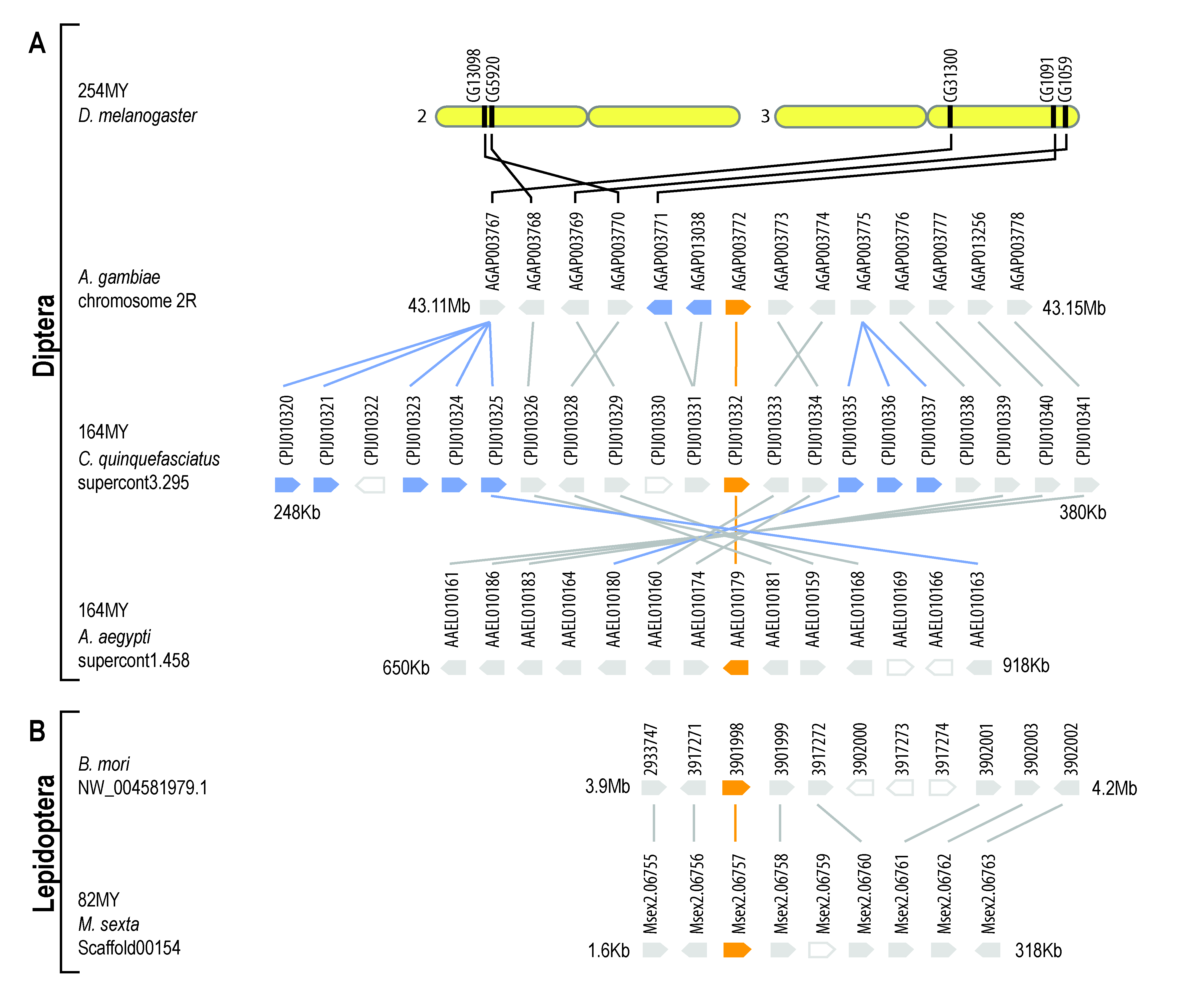

Supplement: Supplementary file 5 — Micro-synteny around NOX4-art gene (orange arrow) in Diptera and Lepidoptera. Orthologous genes are represented by grey arrows, paralogs by blue arrows and genes where no orthologous or paralogous relationship could be determined within the genomes are depicted in grey-wired arrows. A) Complete chromosomes 2 and 3 of Drosophila melanogaster and scaffold/chromosome regions, where NOX4-art was found, in Anopheles gambiae, Culex quinquefasciatus and Aedes aegypti. B) Scaffold regions, where NOX4-art was found, in Bombyx mori and Manduca sexta. Estimated divergence between Anoheles gambiae and other diptera species and the two lepidopteran species are given in million years (MY) [57]. (TIFF 1688 kb) [file 12862_2017_940_MOESM5_ESM.tif]
